# Supplementary material for: Redox Chemistry of the Subphases of α-CsPbI2Br and β-CsPbI2Br: Theory Reveals New Potential for Photostability
Source: Nanomaterials (Basel). 2023 Jan 9;13(2):276. doi: 10.3390/nano13020276 (PMC9862745; doi:10.3390/nano13020276)
Supplement: Supplementary file 1 [file nanomaterials-13-00276-s001.zip › nanomaterials-2151481-supplementary.pdf]

Redox Chemistry of the Subphases of α-$CsPbI_2Br$ and β-$CsPbI_2Br$: Theory Reveals New Potential for Photostability

Lavrenty Gennady Gutsev [1,2,*], Sean Nations [1], Bala Ramu Ramachandran [1], Gennady Lavrenty Gutsev [3], Shengnian Wang [1], Sergei Aldoshin [2] and Yuhua Duan [4]

[1] Institute for Micromanufacturing, Louisiana Tech University, Ruston, LA 71272, USA
[2] Federal Research Center for Problems of Chemical Physics and Medicinal Chemistry of RAS, Semenov Prospect 1, Chernogolovka 142432, Russia
[3] Department of Physics, Florida A&M University, Tallahassee, FL 32307, USA
[4] National Energy Technology Laboratory, United States Department of Energy, Pittsburgh, PA 15236, USA
* Correspondence: lgutsev@latech.edu

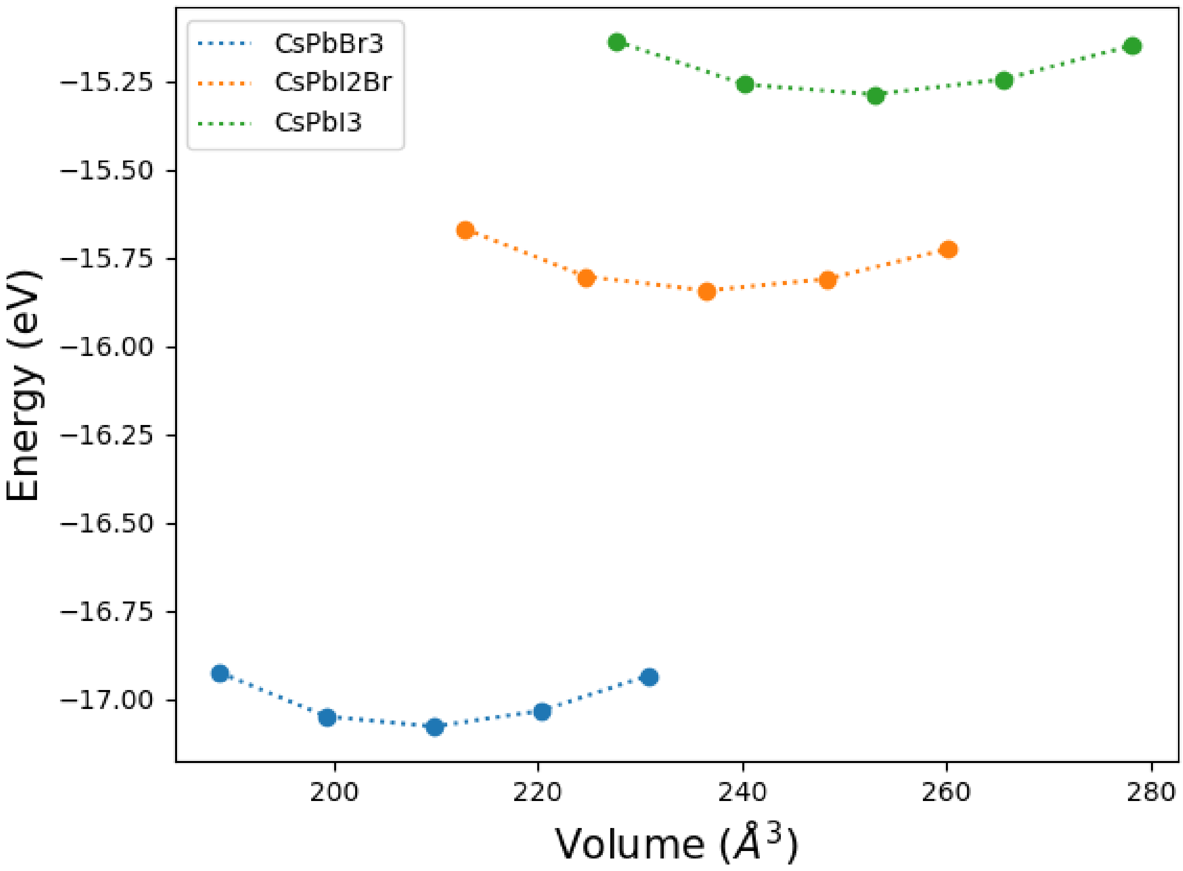

Figure S1. Energy versus volume (EV) curves for α-$CsPbI_3$, α-$CsPbI_2Br$ and α-$CsPbBr_3$.

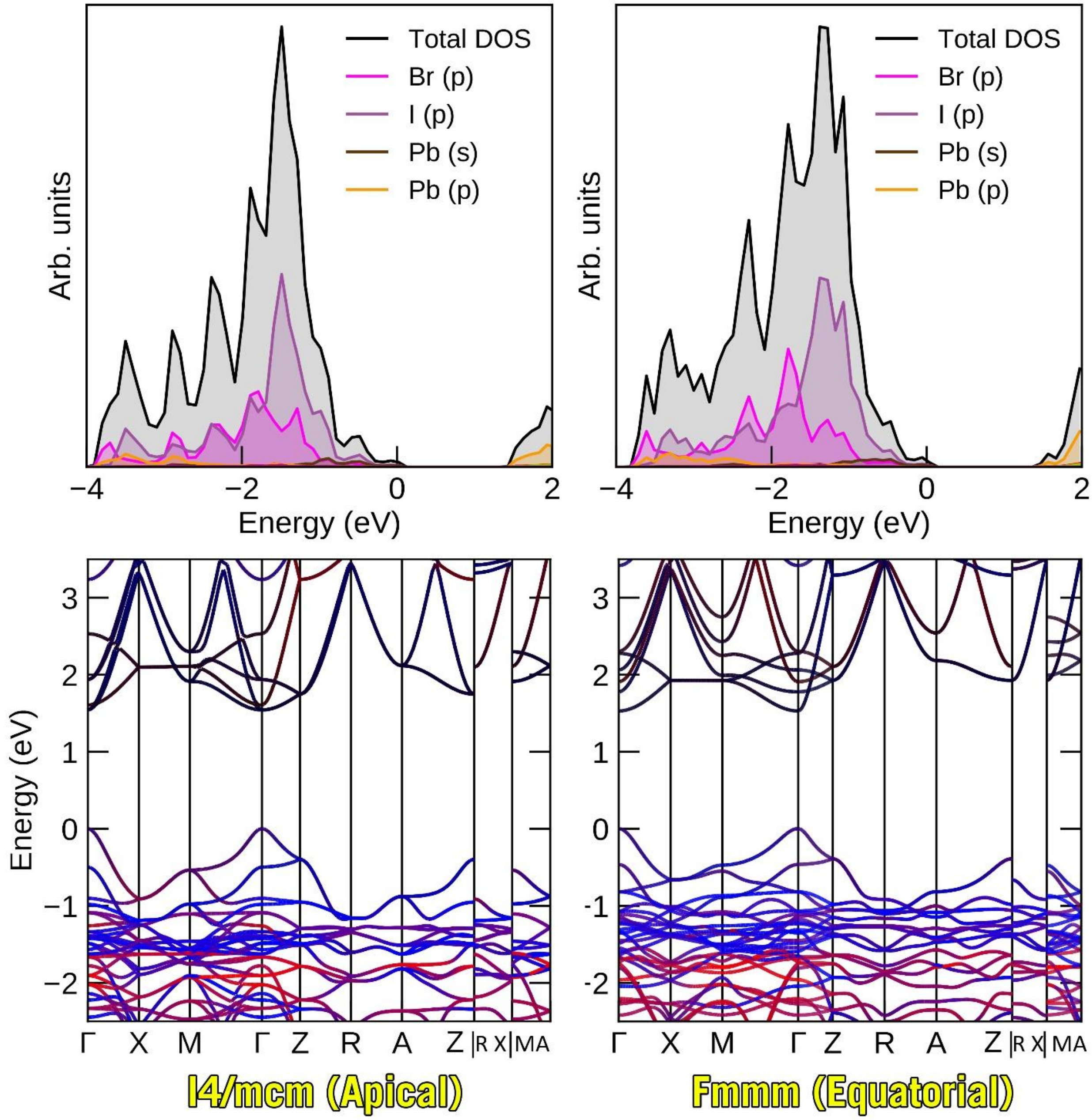

Figure S2. The partial Density of States and band structure of β-CsPbI2Br *I4/mcm* (apical,left) and β-CsPbI2Br *Fmmm* (equatorial). The bromine-based contributions to band structure are in red, iodine in blue. The “darkness” of the conduction band is explained by its majority Pb-p character.

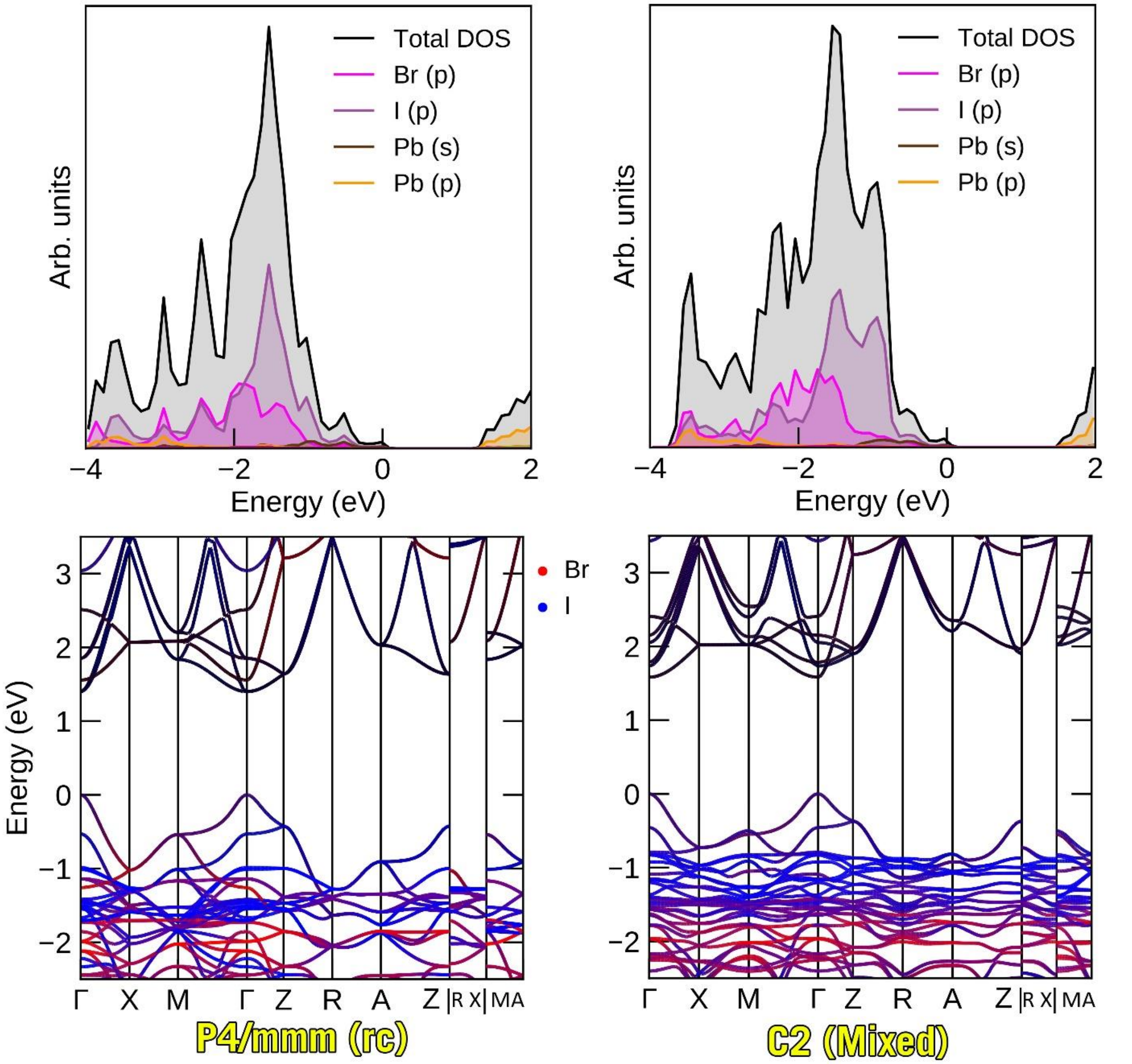

Figure S3. The partial Density of States and band structure of α-$CsPbI_3$ *P4/mmm* (rotated coordinates, left) and β-CsPbI2Br *C2*(mixed). The bromine-based contributions to band structure are in red, iodine in blue. The “darkness” of the conduction band is explained by its majority Pb-p character.

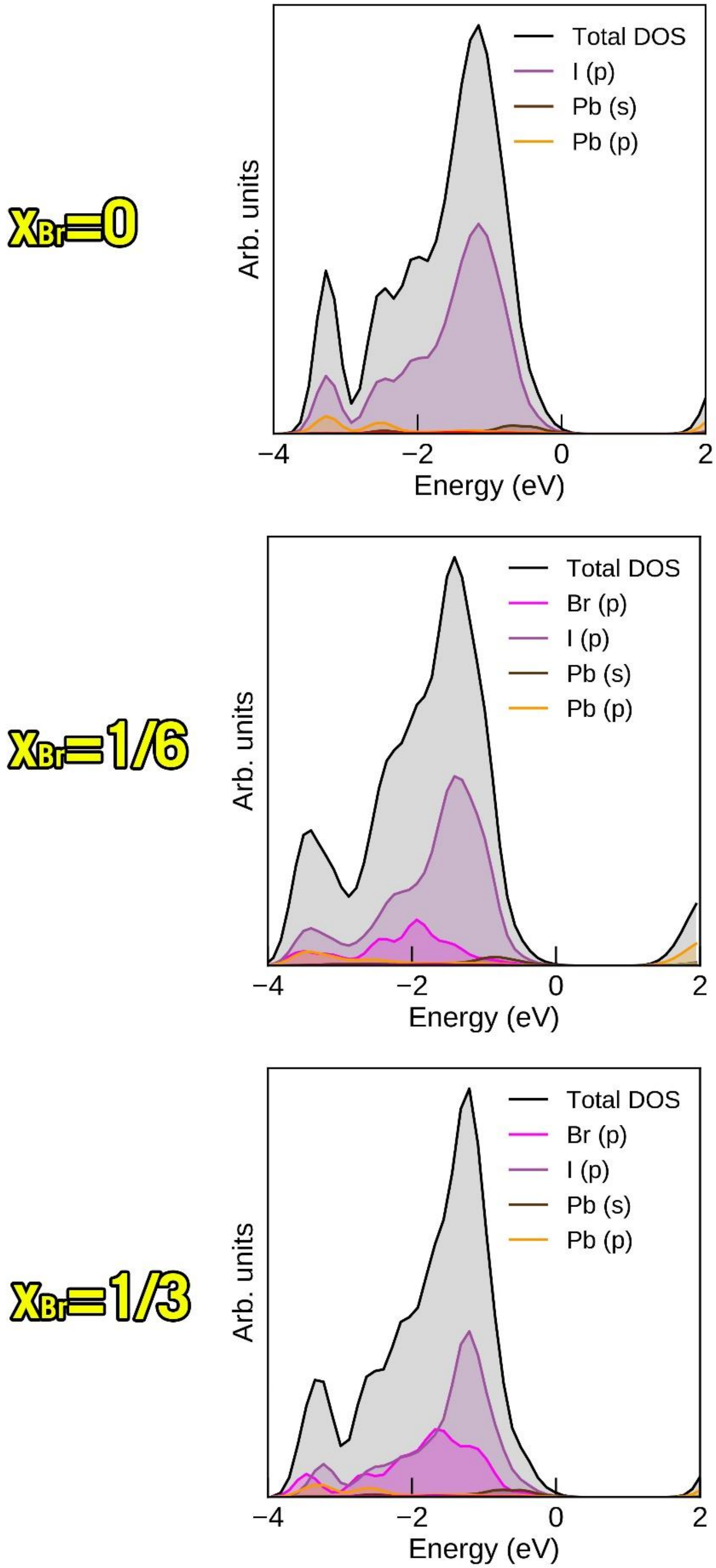

Figure S4. The partial Density of States of α-$CsPb(I_{1-x}Br_x)_3$ x=0,1/3,1/6

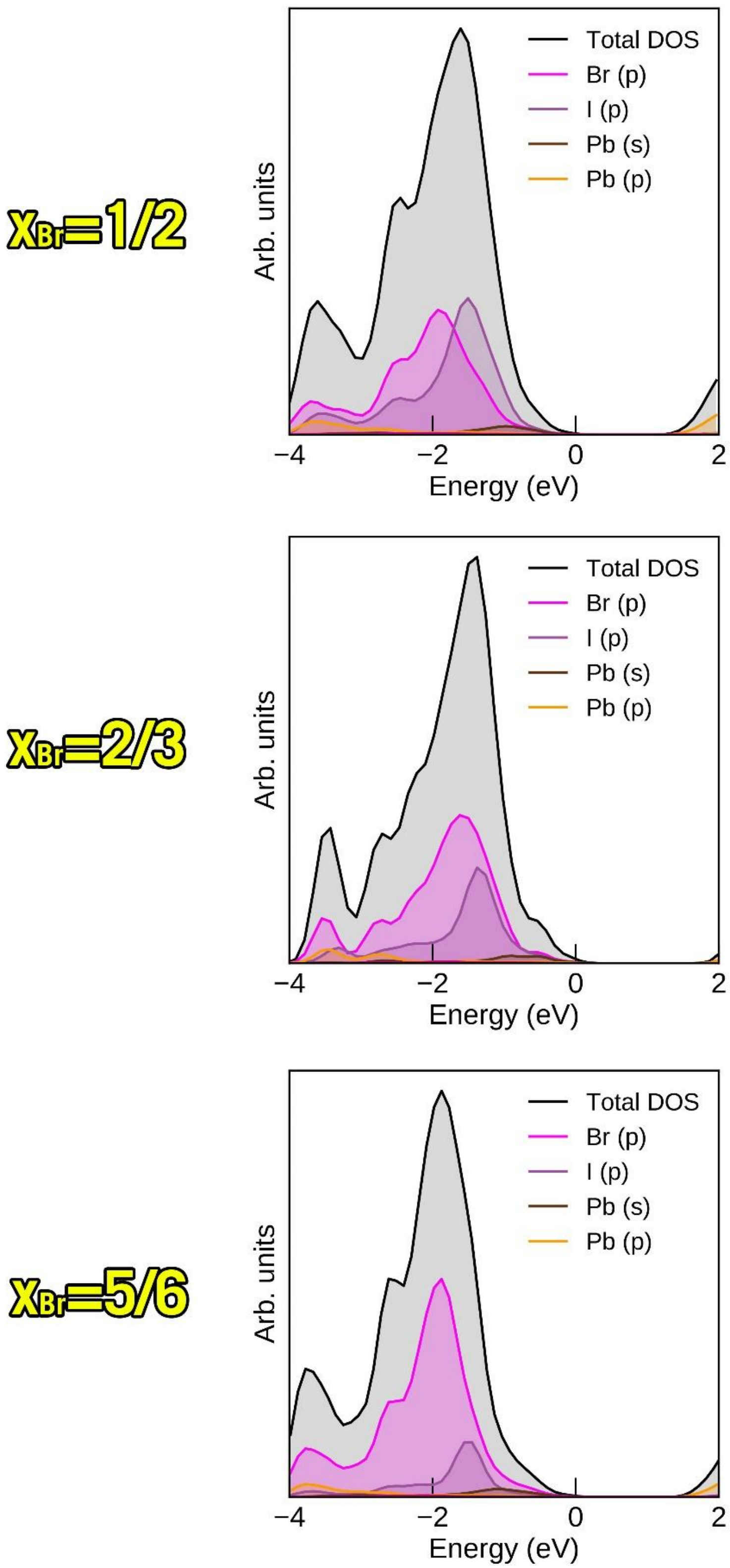

Figure S5. The partial Density of States of α-$CsPb(I_{1-x}Br_x)_3$ x=1/2,2/3,5/6

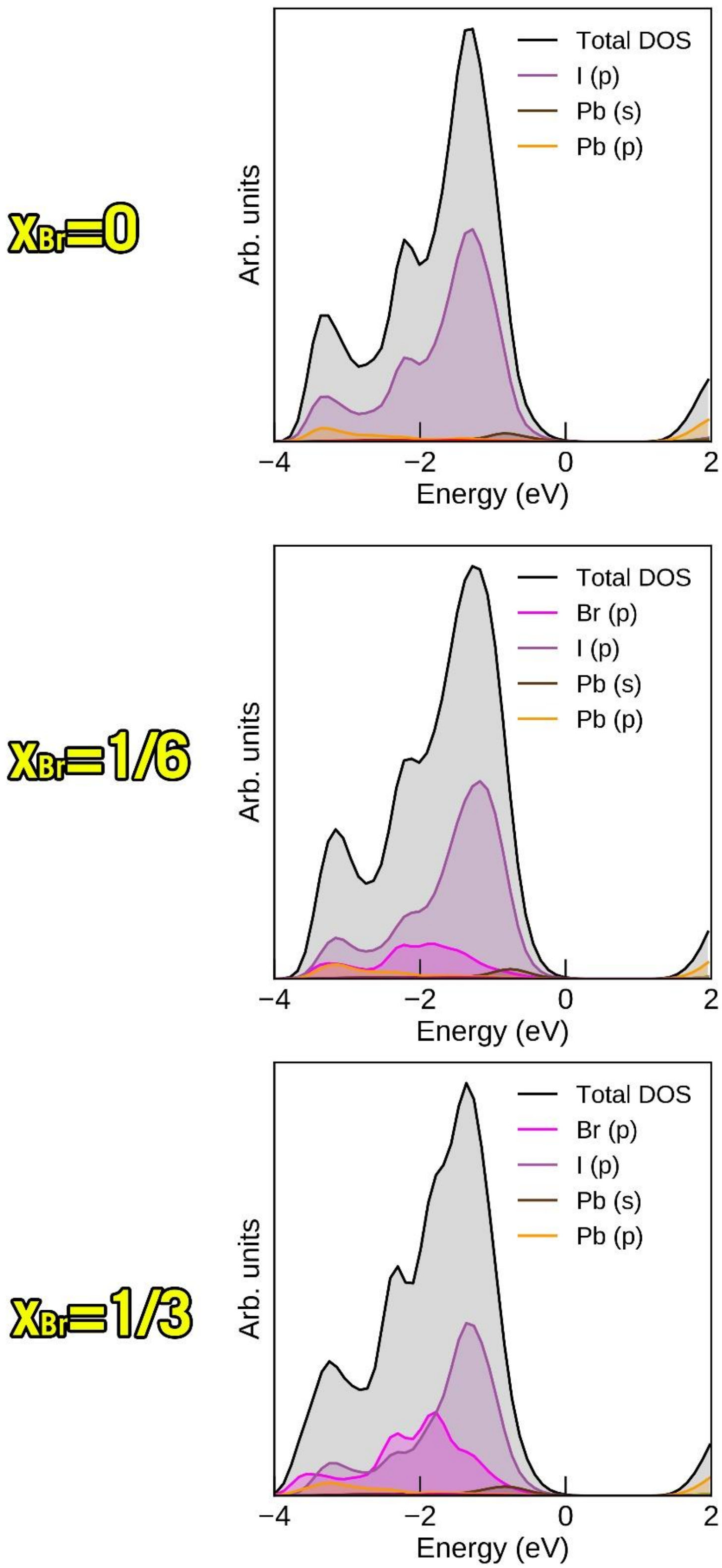

Figure S6. The partial Density of States of β-$\mathrm{CsPb(I_{1-x}Br_x)_3}$ x=0,1/3,1/6

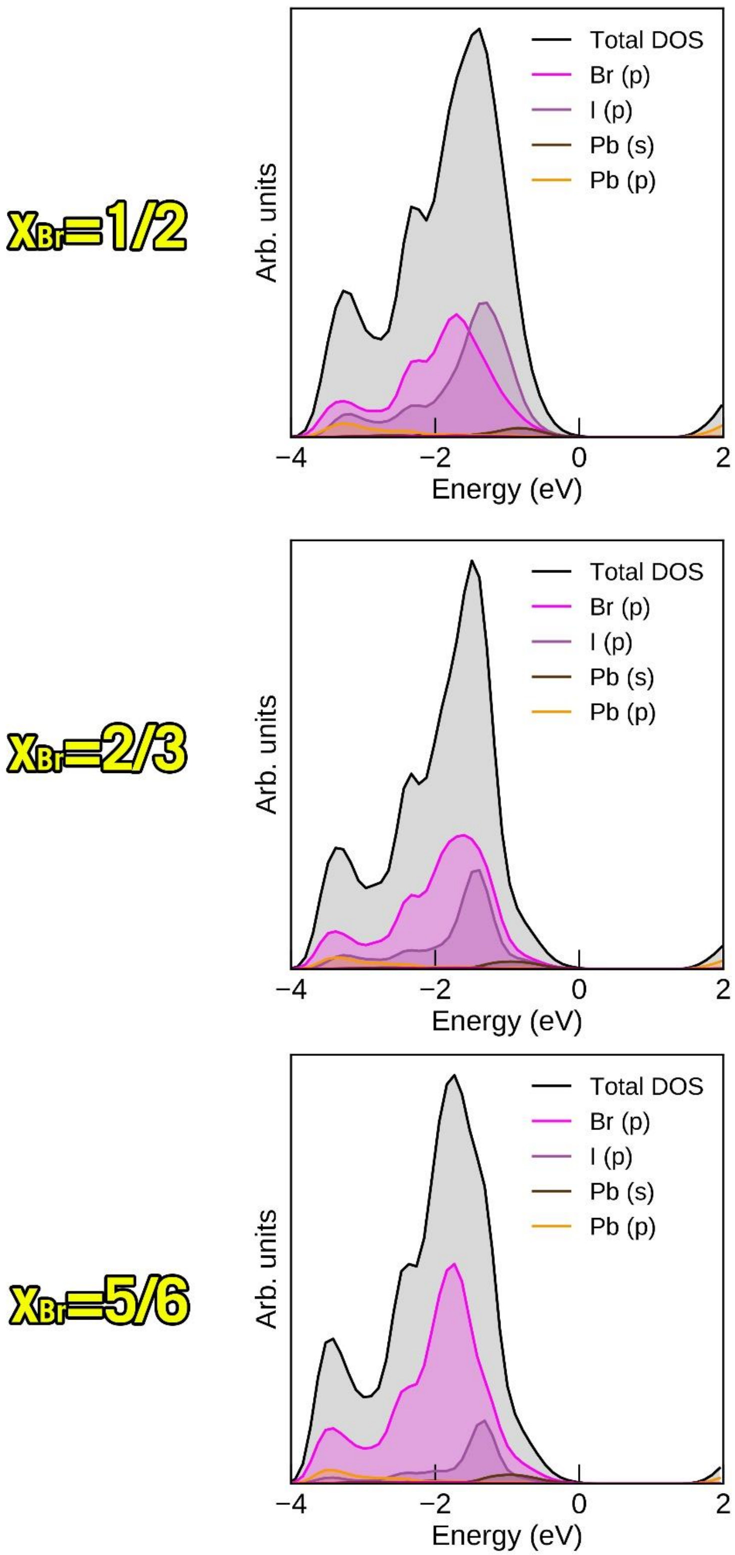

Figure S7. The partial Density of States of β-$\mathrm{CsPb(I_{1-x}Br_x)_3}$ x=1/2,2/3,5/6

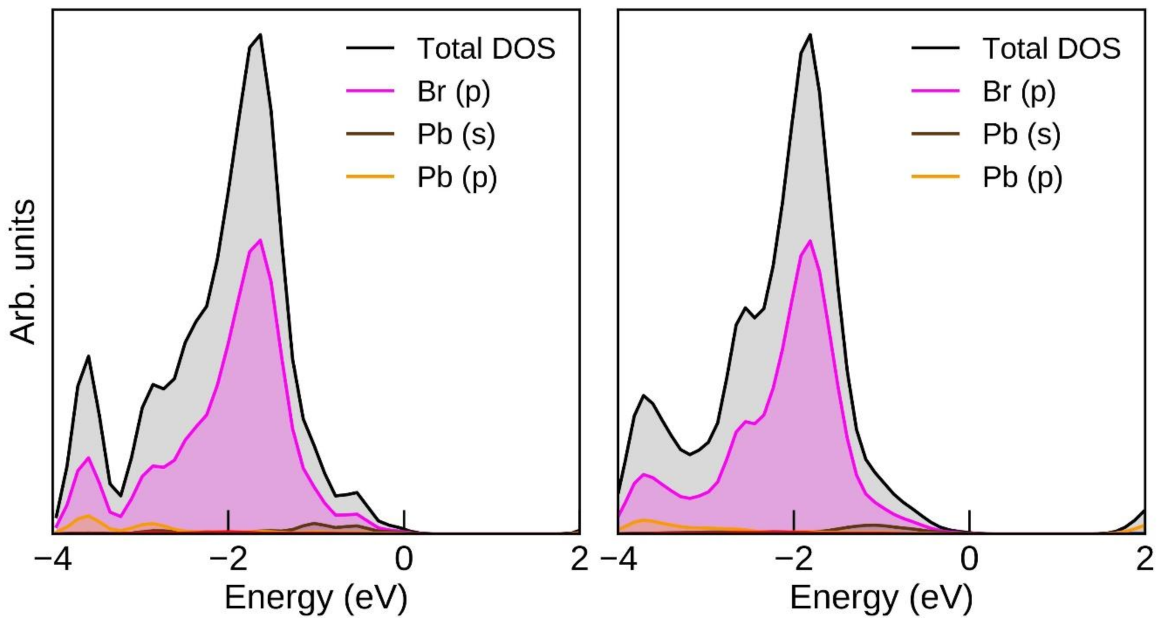

Figure S8. The partial Density of States of α-$CsPbBr_3$ (left) and β-$CsPbBr_3$ (right)

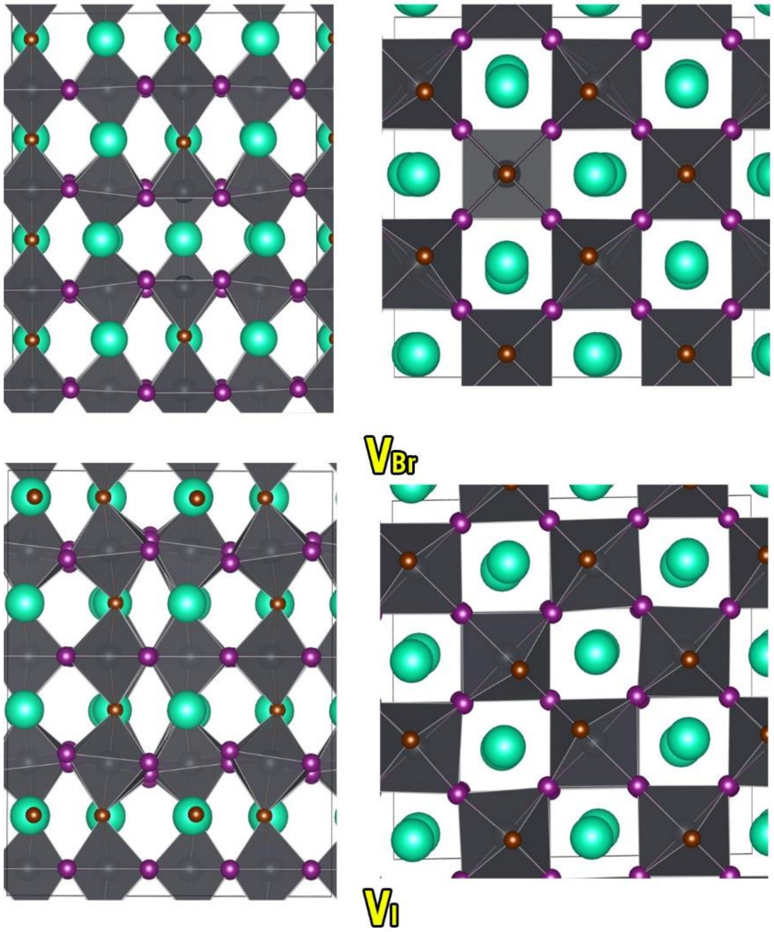

Figure S9 The geometrical distortions caused by bromine and iodine vacancies in α-CsPbI2Br *P4/mmm*
